# Advances in omics-based methods to identify novel targets for malaria and other parasitic protozoan infections

**DOI:** 10.1186/s13073-019-0673-3

**Published:** 2019-10-22

**Authors:** Annie N. Cowell, Elizabeth A. Winzeler

**Affiliations:** 1 Division of Infectious Diseases and Global Health, Department of Medicine, University of California, San Diego, Gilman Drive, La Jolla, CA 92093 USA; 2 Division of Host–Microbe Systems and Therapeutics, Department of Pediatrics, University of California, San Diego, Gilman Drive, La Jolla, CA 92093 USA

**Keywords:** Genomics, Malaria, Plasmodium, Drug discovery, Target discovery, Resistance

## Abstract

A major advance in antimalarial drug discovery has been the shift towards cell-based phenotypic screening, with notable progress in the screening of compounds against the asexual blood stage, liver stage, and gametocytes. A primary method for drug target deconvolution in *Plasmodium falciparum* is in vitro evolution of compound-resistant parasites followed by whole-genome scans. Several of the most promising antimalarial drug targets, such as translation elongation factor 2 (eEF2) and phenylalanine tRNA synthetase (PheRS), have been identified or confirmed using this method. One drawback of this method is that if a mutated gene is uncharacterized, a substantial effort may be required to determine whether it is a drug target, a drug resistance gene, or if the mutation is merely a background mutation. Thus, the availability of high-throughput, functional genomic datasets can greatly assist with target deconvolution. Studies mapping genome-wide essentiality in *P. falciparum* or performing transcriptional profiling of the host and parasite during liver-stage infection with *P. berghei* have identified potentially druggable pathways. Advances in mapping the epigenomic regulation of the malaria parasite genome have also enabled the identification of key processes involved in parasite development. In addition, the examination of the host genome during infection has identified novel gene candidates associated with susceptibility to severe malaria. Here, we review recent studies that have used omics-based methods to identify novel targets for interventions against protozoan parasites, focusing on malaria, and we highlight the advantages and limitations of the approaches used. These approaches have also been extended to other protozoan pathogens, including *Toxoplasma*, *Trypanosoma*, and *Leishmania* spp., and these studies highlight how drug discovery efforts against these pathogens benefit from the utilization of diverse omics-based methods to identify promising drug targets.

## Background

Protozoan parasitic infections cause significant morbidity and mortality worldwide. Malaria, the most well-known protozoan infection, is caused by parasites from the *Plasmodium* genus, with *P. falciparum* and *P. vivax* causing the majority of cases. The parasites are transmitted as sporozoites by mosquitoes into the host’s bloodstream, before invading liver cells and undergoing a rapid growth and division phase as schizonts [1]. The liver cells eventually rupture, releasing these parasites into the bloodstream as nonmotile merozoites, to begin the asexual stage of infection. A subset of asexual blood-stage parasites subsequently develops into male and female gametocytes, which can be picked up by mosquitoes and transmitted to other hosts.

There were an estimated 219 million cases of malaria and 435,000 malaria-related deaths worldwide in 2017 [2], with most cases occurring in sub-Saharan Africa and the majority of deaths in children younger than 5 years old. A fully protective vaccine is not available, so malaria prevention is primarily achieved through the use of bed nets and insecticides. Malaria treatment utilizes small-molecule drugs, with the major drug classes (Table 1) including the following: 4-aminoquinolines, which interfere with heme detoxification; 8-aminoquinolones, whose mechanism is unknown; aryl amino-alcohols, which are thought to interfere with heme detoxification; antifolate drugs, which inhibit folate synthesis; antibiotics, which inhibit protein synthesis; napthoquinones, which inhibit the cytochrome bc_1_ complex; and artemisinin compounds, whose target is unclear but involves the parasite stress response. Artemisinin compounds are an important component of first-line treatment for *P. falciparum* malaria in the majority of countries around the world. However, a major threat to malaria control is resistance to antimalarial medications.

**Table 1:** Overview of the protozoan pathogens highlighted in this review

Pathogen	Disease(s) caused	Current treatments	Mechanism of action
*Plasmodium falciparum*	Malaria	4-Aminoquinolines (chloroquine, amodiaquine, piperaquine)	Inhibit heme detoxification
		8-Aminoquinolones (primaquine, tafenaquine)	Unknown
		Aryl amino-alcohols (lumefantrine, mefloquine)	Inhibit heme detoxification
		Antifolate drugs (proguanil, pyrimethamine, sulfadoxine)	Inhibit folate synthesis
		Antibiotics (doxycycline, clindamycin)	Inhibit protein synthesis
		Napthoquinones (atovaquone)	Inhibit cytochrome bc_1_ complex
		Artemisinin compounds (artemisinin, artemether, dihydroartemisinin)	Oxidative stress
*Trypanosoma*	Chagas disease	Nitroheterocyclic drugs (nifurtimox, benznidazole)	Oxidative stress
	Sleeping sickness	Pentamidine	Disrupts mitochondrial processes
		Melarsoprol	Inhibits trypanosomal redox metabolism and glycolysis
		Suramin	Disrupts trypanosomal redox metabolism and glycolysis
		Eflornithine	Inhibition of ornithine decarboxylase
*Leishmania*	Cutaneous, visceral, or mucosal leishmaniasis	Pentavalent antimonial compounds	Unclear
		Amphotericin B	Targets the main parasite membrane sterol
		Miltefosine	Interferes with cell membrane composition
		Paromomycin	Inhibits protein synthesis
*Toxoplasma*	Flu-like illness, disseminated infection, congenital infection	Pyrimethamine	Inhibit folate synthesis
		Sulfadiazine	

For more detailed information on treatments, mechanisms of action, and mechanisms of resistance for each pathogen, please refer to the following literature: *P. falciparum* [3], *Trypanosoma* [4–7], *Leishmania* [8–11], and *Toxoplasma* [12, 13]

**Fig. 1:**
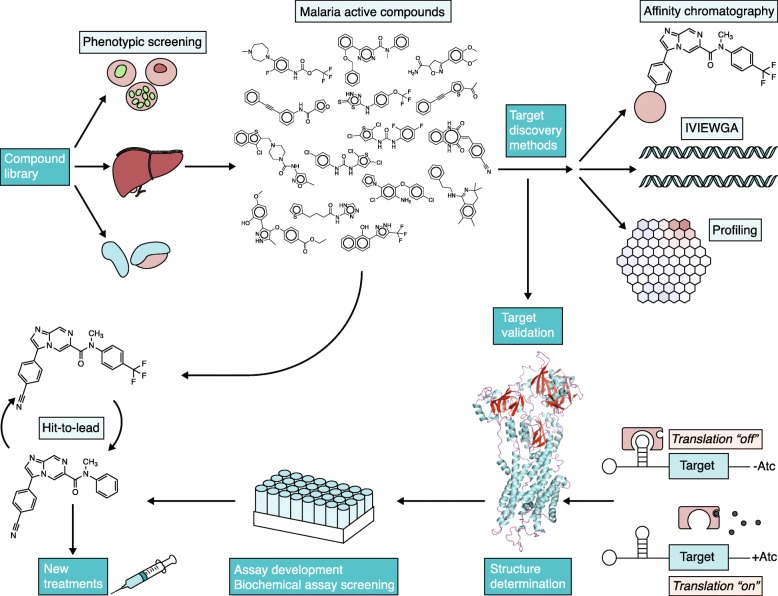
Overview of the antimalarial target discovery and drug discovery processes. Phenotypic screening is undertaken with diverse compound libraries using assays that target different stages of the malaria life cycle: blood stage, liver stage, and gametocytes. Compounds that demonstrate potent antimalarial activity can go directly into hit-to-lead studies and can progress to clinical studies. Simultaneously, target discovery can be carried out using different methods, such as affinity chromatography, in vitro evolution and whole-genome analysis (IVIEWGA) [17], and metabolic profiling. Target validation can be carried out using gene knockdown approaches such as the TetR-aptamer system [18]. Genome-wide essentiality data can also help with target validation. Target structures can then be determined, and recombinant protein targets can be used in biochemical screens. Hit-to-lead optimization can occur without knowing a target, although development is facilitated when the target is known

Owing to continual issues with antimalarial drug resistance, there is an ongoing need to place new molecules in the development pipeline. Emerging artemisinin resistance presents a major current threat to global health [14, 15]. The availability of the major *Plasmodium* genome sequences, combined with improvements in parasite culture adaptation and animal models of infection, have enabled the identification of novel drug targets and have improved our understanding of the host and parasite factors that contribute to infection. Another major advance in antimalarial drug discovery has been a shift towards cell-based phenotypic screening, which identifies changes in phenotype that occur following the exposure of whole microorganisms or cells to drug candidates. This strategy contrasts with single-enzyme screening, which focuses on the screening of compounds against a single potential target enzyme (reviewed in [16]) (Fig. 1). For cell-based phenotypic screening, prior knowledge of the drug target is not necessary, novel targets can be identified, and compounds that do not permeate the cell membrane are rapidly eliminated.

Extremely large compound libraries have been screened for promising antimalarial compounds, primarily using *P. falciparum* strains that have been adapted to culture [19–22]. There have been more recent advances in developing new methods for *P. vivax* and for specific parasite stages, such as the asexual and gametocyte blood stages and the liver stage. The compounds identified using cell-based phenotypic screening approaches can be the starting points for drug discovery, and scaffold series (core chemical structures) arising from phenotypic screens have filled the antimalarial drug development pipeline for the past decade.

Although drug development can be accomplished without knowing how a compound works in the cell, hit-to-lead optimization (during which small molecule hits from a high-throughput screen undergo optimization in order to identify promising lead compounds) is greatly facilitated if the target is known. Following the phenotypic screening of compounds against *P. falciparum* parasites at multiple life cycle stages (the asexual blood stage, liver stage, and gametocytes), compounds that demonstrate potent activity can go directly into hit-to-lead studies, which can then continue to clinical studies (Fig. 1). To identify the target of the compound (a process called target deconvolution), affinity chromatography, in vitro evolution and whole-genome analysis (IVIEWGA) [17], and metabolic profiling can be performed. Target validation can then be carried out using gene knockdown approaches such as the TetR-aptamer system [18]. Genome-wide essentiality data can also help with this step. The target structure can then be determined and further optimized with high-throughput screening. Powerful tools such as structure-guided drug discovery, fragment screening, and DNA-encoded libraries can be used if good targets are available. A good target is one that is critically essential, such that an incomplete knockdown results in parasite death. Ideally, it would also have a pocket or catalytic site that will accommodate a small molecular inhibitor. Targets discovered using deconvolution are considered “chemically validated” and are thus of higher value as these are more likely to have pockets that accommodate small molecules, and if inhibition can be achieved at physiologically relevant concentrations, inhibition of such targets could potentially lead to parasite death and, in the best cases, patient cure.

Here, we highlight recent studies that have used omics-based methods to identify novel targets for parasitic protozoan infections, with a focus on malaria. We review recent advances in parasite genomic, proteomic, transcriptomic, and epigenomic methods that have been used to generate functional genomic and omic data that provide a foundation for target deconvolution. We also examine studies of host genetics, transcriptomics, and genomics that have analyzed the host response to malaria infection. In addition, we briefly discuss major advances in target identification using omics-based methods in other protozoan pathogens, including *Toxoplasma*, *Trypansoma*, and *Leishmania*. Many promising novel targets have been identified for these pathogens, some of which are conserved across species. Forward genetics approaches have primarily identified proteins that have also been found to be druggable in other species. These targets include translation elongation factor 2 (eEF2), phenylalanine tRNA synthetase (PheRS), cytoplasmic isoleucine tRNA synthase (IRS), lysyl tRNA synthase, P-type cation-ATPase PfATP4, dihydroorotate dehydrogenase, and cytochrome bc1 in *Plasmodium*, in addition to proteasome subunits for *Plasmodium*, *Trypanosoma*, and *Leishmania*, and cyclin-dependent kinase 12 (CDK12) for *Leishmania*.

## In vitro evolution and whole-genome scans for target discovery

A primary method that has been used for target discovery is in vitro evolution and whole-genome analysis (IVIEWGA; reviewed in [17, 23]; Fig. 1; Table 2). In this method, *P. falciparum* parasites are exposed to sub-lethal levels of compounds until resistant parasites are produced. The genomes of the resistant parasites are compared to their isogenic parent parasite in order to identify mutations that arose during the process of resistance acquisition. This method generates hypotheses about drug resistance mechanisms and about the potential drug target that can be validated with further testing and can thus enable the design of improved therapies. A limitation of this method is that if a mutation is identified in an uncharacterized gene, time-consuming studies may be required to understand whether the gene is a drug target or a drug resistance gene, or whether the mutation is merely a background mutation.

**Table 2:** Summary of omics-based technologies used for target discovery and validation for protozoan pathogens

Technology	Tools used	Application	Advantages	Disadvantages
In vitro evolution and whole-genome analysis	- Tiling microarrays - Whole-genome sequencing	- Identifying the targets of compounds - Analyzing the mechanisms of resistance	- Can determine the targets of compounds from phenotypic screens - Simultaneously enables the assessment of mechanisms of resistance - High specificity	- Resistance mutations may obscure mutations in the gene encoding the target - Inability to generate in vitro resistance to some compounds, particularly fast-killing compounds
Genome-wide essentiality studies	- *piggyBac* transposon system - Targeted barcode gene knockouts	- Determining essential pathways, thereby identifying potentially druggable pathways	- Assesses the entire genome at once - A gene or pathway can be directly linked to a particular phenotype	- Transposition may occur in essential genes - Assessment is limited to annotated genes - Some genes are more amenable to transposon mutagenesis or gene knockout
Transcriptomic analysis	- RNA-seq of the pathogen - Dual RNA-seq of host cells and the pathogen	- Identifies pathogen gene pathways that are upregulated during infection - Identifies host pathways that are important in response to infection	- Provides information about the upregulation of genes relative to other pathways	- Assessment is limited to annotated genes - High sequencing coverage is needed to detect meaningful changes resulting from low-level infection
Proteomics	- Mass spectrometry - Nuclear magnetic resonance-based structure guidance	- Target identification - Target validation - Understanding mechanisms of resistance	- Can identify the cellular location of a target or mechanism of resistance - Targeting protein-protein interactions increases the number of potential inhibitor-binding locations	- Multiple proteins are typically identified in initial studies - Success is dependent on whether the inhibition is potent enough to cure the disease
Epigenomics	- ATAC-seq (assay for transposase-accessible chromatin using sequencing) - ChIP-seq (chromatin immunoprecipitation sequencing)	- Identifying which genes are expressed or silent at different stages of the parasite life cycle	- Can help to interpret whole-genome data by assessing whether intergenic mutations are in regulatory regions	- ATAC-seq is biased against AT-rich sequences

This approach has been used recently to identify or confirm several novel promising antimalarial targets, including eEF2 [24]; PheRS [22]; the proteasome [25], a homolog of mammalian cleavage and polyadenylation specificity factor subunit 3 (PfCPSF3) [26]; and the bifunctional farnesyl/geranylgeranyl diphosphate synthase (PfFFPS/GGPPS) [27] (Table 3).

**Table 3:** Potential target proteins and pathways identified in recent studies of protozoan pathogens

Parasite	Potential target	Pathway	Parasite stage(s) at which target can be inhibited	Function
*Plasmodium*	Translation elongation factor 2 [24]	Protein synthesis [28]	Blood, liver, gametocytes	Mediates GTP-dependent ribosome translocation along mRNA [28]
	Phenylalanine tRNA synthetase [22]	Protein translation	Blood, liver, gametocytes	Catalyzes the attachment of amino acids to tRNA [29]
	β2 subunit of the proteasome [30]	Ubiquitin-proteasome pathway	Blood, liver, gametocytes	Catalyzes protein degradation [31]
	Farnesyltransferase [32]	Enables cell cycle progression	Blood, liver	Adds a farnesyl group to the carboxyl terminus of specific proteins [33]
	Dipeptidyl aminopeptidase 1 [32]	Hemoglobin catabolism	Blood	Cleaves amino acids from proteins or oligopeptides [34]
	Aminophospholipid-transporting P-type ATPase [32]	Maintenance of cell membrane potential	Blood, liver	Phospholipid transport [35]
	Thymidylate synthase [32]	DNA synthesis	Blood	Folate biosynthesis
	Cyclic GMP-dependent protein kinase [36, 37]	Phosphorylation-dependent signaling	Blood, gametocytes	Enables parasite egress from and invasion of red blood cells [38]
	Calcium-dependent protein kinase 5 [36]	Calcium regulation	Blood	Critical for parasite egress from red blood cells [39]
	Cyclin-dependent-like kinase 3 [40]	Gene expression	Blood, liver, gametocytes	Plays a role in mRNA splicing [40]
*Trypanosoma*	β4 subunit of the proteasome [41]	Ubiquitin-proteasome pathway	Tissue and blood stage	Catalyzes protein degradation
	Peroxin interaction [42]	Glycosomal biogenesis and import	Blood stage	Enables glucose metabolism
*Leishmania*	β4 subunit of the proteasome [41]	Ubiquitin-proteasome pathway	Intracellular	Catalyzes protein degradation
	Cyclin-dependent kinase 12 [43]	Control of transcription and cell division [44]	Intracellular	Precise role unclear
*Toxoplasma*	Claudin-like apicomplexan microneme protein [45]	Formation of tight junctions through which parasites enter host cells	Tachyzoites	Essential for the invasion of host cells

The method can have a high degree of specificity. For example, Kato and colleagues [22] investigated the bicyclic azetidine BRD3444 and found high-quality nonsynonymous single-nucleotide variants (SNVs) that localized to the alpha subunit of PheRS. Xie and colleagues [25] verified that the target of bortezimib, a proteasome inhibitor, localizes to subunit β5 of the proteasome using this method. A comprehensive analysis of mutations that arose in 262 *P. falciparum* whole-genome sequences from parasites that were resistant to at least 1 of 37 diverse compounds identified several new promising target-inhibitor pairs [32]. For mutations that were identified in genes encoding enzymes, where docking and homology modeling confirmed that the mutations were located in the active site, these enzymes were considered promising potential targets. These included farnesyltransferase, dipeptidyl aminopeptidase 1, aminophospholipid-transporting P-type ATPase (previously named PfATP2), and thymidylate synthase portion of the dihydrofolate-reductase-thymidylate synthase enzyme.

## Proteomic methods for target deconvolution

A problem with using IVIEWGA is that if there is a clearly identifiable resistance gene, mutations in this gene may appear repeatedly in resistant parasites, obscuring the true target and the mechanism of action of the compound. For example, in vitro evolution has failed to identify the target of one of the most advanced compounds in the antimalarial pipeline, the imidazopiperazine ganaplacide (KAF156), repeatedly revealing membrane-based transporters, such as PfCARL, that are nonessential and confer resistance to multiple compounds [46]. In such cases, the next best strategy is proteomics (reviewed in [47]; Table 2).

Two basic approaches involving protein capture are available: covalent methods (in which some prior knowledge of the target is needed, using capture agents that will demonstrate specific binding with a particular compound) and noncovalent methods (where prior identification of the precise target is not required). Noncovalent chemoproteomic methods were used to identify phosphatidylinositol-4-kinase (PI4K) as the target of another compound in the antimalarial pipeline, MMV390048, although IVIEWGA was also used to support that conclusion [48]. Covalent methods have been used to show binding between compounds and *P. falciparum* proteasome subunits [30, 49]. In other protozoan species, noncovalent, competition approaches have been used to assist with target discovery [43], as discussed further below. Proteomics can be powerful, but a general problem with the approach is that multiple proteins are usually identified, and thus determining the correct target can be challenging and may require time-consuming follow-up studies. However, as in the case of MMV390048, supplemental genetic or genomic data can help to confirm a potential target [47].

The genetic and mechanistic basis of *P. falciparum* artemisinin resistance is an area of intense study in the malaria community and one that has benefited greatly from genetic and genomic methodologies, such as genome-wide association studies (GWAS) and IVIEWGA (reviewed in [50]). Nevertheless, the mechanism of resistance remains poorly defined and proteomics approaches have been used to try to elucidate this further. Previously, the Haldar group [51] showed that the *kelch13* C580Y mutation, which confers artemisinin resistance, results in decreased binding to and decreased ubiquitinylation-dependent proteosomal degradation of *P. falciparum* phosphatidylinositol-3-kinase (PfPI3K). PfPI3K phosphorylates phosphoinositol at the 3′ position to yield phosphatidylinositol-3-phosphate (PI3P), a phospholipid that is involved in recruiting proteins to membranes. Thus, the C580Y mutation results in increased levels of PI3P.

More recently, the same group sought to use proteomics to further characterize the role of PI3P in artemisinin resistance [52]. Because prior studies had suggested that PI3P helps to bring the exported virulence factor *P. falciparum* erythrocyte membrane protein 1 (PfEMP1) to the surface of the infected red cell [51], they used whole-genome-derived proteomic data to tie Kelch13 to PfEMP1. Specifically, they performed mass spectrometry of immunoprecipitates obtained with a PfEMP1 antibody and identified 503 proteins that were detected in both of the 2 experimental replicates. This set of proteins was enriched for those involved in translation and protein trafficking, including Kelch13. The C580Y mutation in *kelch13* resulted in an increase in PI3P tubules and vesicles. These data are intriguing and provide further information about how the PI3P lipid mitigates the deleterious effects of artemisinin on the parasite. Nevertheless, it is important to remember that, in general, mass spectrometry data are biased towards the most abundant cytoplasmic proteins (such as those involved in translation and glycolysis). Without normalizing to mass spectrometry data from immunoprecipitation pulldowns with other antibodies or to whole-genome-derived proteomic data, possible artifactual associations may be revealed with immunoprecipitations. In addition, probability values need to be adjusted for multiple hypothesis testing to minimize high false-positive and false-negative rates when dealing with large genome-scale numbers [53].

## Advances in high-throughput phenotypic screening approaches

The use of in vitro evolution to identify antimalarial targets has depended on the identification of compounds that have antimalarial activity. Although many of the chemical compounds used in recent studies were identified in large-scale phenotypic screens with *P. falciparum* asexual blood stages [20, 21, 54], there has been recent progress focused on other stages of the malaria parasite life cycle, including gametocytes, liver stages, and hypnozoites. Many of the drugs that are currently in use do not appear to prevent the spread of parasites from individuals with malaria to mosquitoes, because these drugs (for example, chloroquine) appear to be inactive against metabolically inactive gametocytes, which are sexual-stage parasites. Plouffe and coworkers [55] developed a screening method to identify compounds that are active against stage V gametocytes, the parasite stage that is responsible for the transmission and spread of malaria, confirming that many antimalarial compounds are unlikely to block the spread of the disease. This approach primarily identified live or dead late-stage gametocytes, whereas more descriptive lower-throughput assays for activity against sexual stages have also been established and used to examine medium-sized chemical compound libraries. Delves and colleagues [56] recently performed a high-throughput screen of around 70,000 compounds against male and female gametocytes and identified 17 compounds with potent gametocidal activity. This study identified novel chemical scaffolds that had not been identified in asexual blood-stage screens, thus demonstrating the value of screening separately against this life cycle stage.

Drugs that have the potential to act against liver-stage parasites and which could provide chemoprophylactic protection are also receiving more attention. An infection is established by the introduction of a small number of parasites, so in theory, there is less potential for the emergence of drug resistance against compounds that act against this stage. Recently, a very large-scale screen was run against malaria liver stages, and this screen discovered thousands of compounds that have the potential to block the development of malaria [57]. This screen, involving more than 500,000 compounds, was performed over a period of 5 years and involved the dissection of hundreds of thousands of mosquitoes that were infected with luciferase-expressing *P. berghei*, a parasite that causes malaria in rodents. The group tested whether the parasite’s invasion of hepatocytes was blocked by drug candidates. Active compounds were subsequently examined for their ability to block *P. falciparum* asexual blood-stage proliferation, and some of those that did were subjected to target discovery. This revealed a number of potential new cytochrome bc1 and dihydroorotate dehydrogenase inhibitors, some of which were confirmed using IVIEWGA methods. This study also identified a number of compounds that might have the potential to work by acting against possible, as yet unknown, human targets. Further studies will be needed to discover the mechanisms of action of these compounds.

## High-throughput genetic validation of targets

**Fig. 2:**
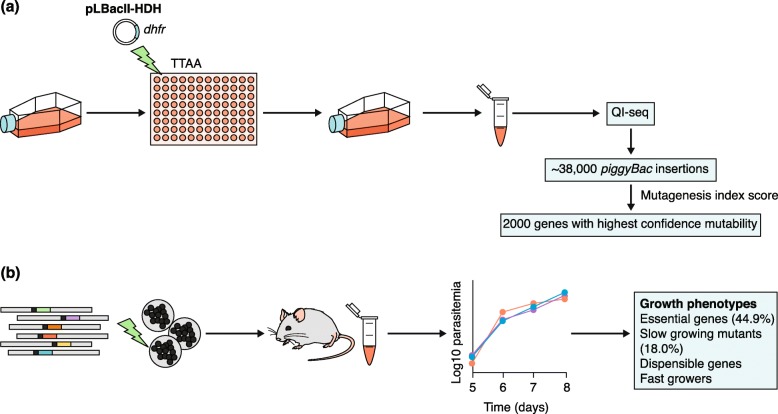
High-throughput genetic validation of targets. Two methods have been used to determine gene essentiality. **a** Zhang et al. [36] used a *piggyBac* transposon system in *P. falciparum* to determine genes that could be disrupted using culture conditions that were considered ideal for the asexual blood stage [36]. Transfection with the *piggyBac* plasmid (pLBacII-HDH) was performed in a 96-well plate, and parasites containing the plasmid marker (*dhfr*) were selected for and regrown in culture. DNA was then extracted and quantitative insertion-site sequencing (QI-seq) was performed to determine the sites of insertion. Mutagenesis index scoring was then used to identify genes with the highest confidence of disruption and nondisruption. **b** Bushell et al. [58] used barcode vectors to determine which genes were essential for asexual blood stage growth using an in vivo system in mice. The vectors were transfected into *P. berghei* schizonts, which were inoculated into mice, and growth was determined by measuring parasitemia on subsequent days of infection. Four growth phenotypes were observed, among which “essential genes” and “slow-growing mutants” were determined to be essential or important for asexual blood stage growth

High-throughput methods such as whole-genome sequencing and proteomics may reveal more than one possible target. In cases where there is ambiguity, genome-wide essentiality data can be very helpful given that targets should be, by definition, essential to parasite life (Table 2). Despite the challenges associated with the culture of malaria parasites and the AT-rich genome of *P. falciparum*, which causes difficulty with mapping sequence reads, tremendous progress has been made recently towards mapping gene essentiality in *P. falciparum* blood stages (Fig. 2).

In a system that is analogous to an approach used in *Saccharomyces cerevisiae* at the beginning of the post-genome era [59], Zhang and colleagues [36] performed mutagenesis with a *piggyBac* transposon system and then sequenced the mutagenized *P. falciparum* cultures to identify genes that could be disrupted, and those that had no transposon insertion events and thus were presumably “essential” and likely to be good drug targets (Fig. 2a). This represents a reverse genetics approach in which phenotypes are assessed after the introduction of gene mutations. The authors showed that no transposons could be detected in 2680 of the 5399 genes encoded by the *P. falciparum* genome, indicating that this group of genes was essential during in vitro asexual blood-stage growth. This group of essential genes contained several that are associated with drug resistance. However, the group also included approximately 1000 genes of unknown function, demonstrating a limitation based on the adequacy of genome annotation. For genes with transposons, a mutant fitness score (MFS) was calculated on the basis of the rate of disappearance of a given transposon tag from cultures. This identified potential drug targets that are in development, including cyclic GMP-dependent protein kinase (PfPKG) and calcium-dependent protein kinase 5 (PfCDPK5) (Table 3).

This study represents an important milestone in mapping essential genes in blood stages of *P. falciparum*, but there could be biases within such data because some genes are more amenable to transposon mutagenesis than others. In addition, in 791 cases, MFS phenotypes were assigned on the basis of a single detected transposition event, with the possibility that a random second site point mutation or indel could be contributing to a slow or dropout growth phenotype. Furthermore, transposition can sometimes occur in essential genes, especially if the event is near the 3′ end of the gene. Genome duplications could also lead to false positives: for example, GTP cyclohydrolase, which has been observed to be duplicated in the *P. falciparum* genome [60], was listed as dispensable with a single transposition event. It is also important to note that the work provided little insight into the essentiality of gene products in other life cycle stages or in vivo growth.

In another approach that is analogous to the efforts in the *S. cerevisiae* community [61, 62], targeted barcoded knockouts for all of the genes encoded by the genome of the rodent model malaria parasite *P. berghei* were also created [58] (Fig. 2b). Rodent malaria parasites have certain advantages over *P. falciparum* in that their genomes are less AT-rich and thus easier to work with. In addition, the liver and transmission stages are more available for experimental investigation in these rodent models than in human infections. However, it is important to mention that only evolutionarily and functionally orthologous genes can be studied with this method. Bushell et al. [58] created a set of 2578 barcoded strains using publicly available knockout vectors with gene-specific molecular barcodes that could be grown en masse in mice. Experiments examining the competitive growth during the asexual blood stage showed phenotypes for two thirds of the strains. This work revealed 1196 genes (45%) that were likely to be essential or important for normal parasite growth, most of which were involved in major basic cellular processes. Known drug targets were identified including dihydroorotate dehydrogenase, which is located in the mitochondria, as well as known drug resistance genes, such as the chloroquine resistance transporter. Potentially druggable pathways that were identified included the glycosylphosphatidylinositol (GPI)-anchored surface protein synthesis pathway, which is a promising drug target in fungi [63], and enzymes in the phosphatidylcholine biosynthesis pathway, which are the speculated targets of the bis-thiazolium drugs [64]. Pathways involved in glycolysis and in mitochondrial maintenance and energy production were also essential for growth. When interpreting this set of data, it is important to note that some pathways that have already been identified as potential drug targets were not shown to be essential. For example, the sphingolipid pathway has been identified as a promising drug target [65], but none of the genes that are involved in this pathway showed essentiality for normal in vitro growth. A possible explanation is that if the parasite is able to scavenge particular substrates from the host cell, then the knockout of genes involved in producing those substrates may not result in impaired growth, although further studies would be needed to investigate this for specific genes. The study is also limited by issues concerning genome annotation. The vector library that was used to perform the knockouts covered approximately half of the protein-coding genome of *P. berghei*; therefore, many genes could not be assessed [58]. In addition, over one third of the genes that were identified lacked known domains or were of unknown function. Finally, it is important to keep in mind that even though a gene might be essential, it may not be “druggable.” Nevertheless, genome-wide essentiality efforts have been very beneficial to the drug discovery community.

## Parasite transcriptomic analysis

Gene expression data can also provide clues about which genes may be drug targets. If a compound acts during specific times in the parasite life cycle, then we might expect the target to be transcribed during this part of the life cycle. Advances in single-cell RNA sequencing (RNA-seq) have allowed a comprehensive examination of transcription throughout the parasite life cycle using the rodent parasite *P. berghei* as a model system [66]. Through the analysis of thousands of single-cell transcriptomes from many different parasite life cycle stages and orthologous gene mapping across species, the authors were able to create a comprehensive gene expression roadmap. These data will be immensely valuable to those seeking to understand the results of forward or population genetics studies. For example, a gene that is transcribed exclusively in oocysts (which develop in mosquitoes) is unlikely to be a target of a compound that is active in blood stages.

**Fig. 3:**
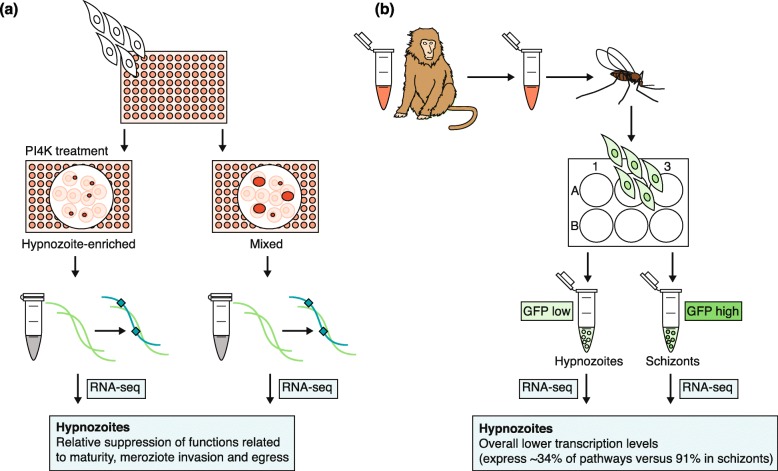
Methods for transcriptional profiling of the *Plasmodium* hypnozoite. **a** Gural et al. [67] used a micropatterned primary human hepatocyte co-culture (MPCC) system to support the growth of *P. vivax* hypnozoites [67]. Cultures were enriched for hypnozoites by treating with a phosphatidylinositol-4-kinase (PI4K) inhibitor, and RNA was then extracted and enriched for *P. vivax* transcripts using biotinylated baits, before being sequenced and compared to RNA from untreated cultures. **b** Voorberg-van der Wel et al. [68] infected rhesus monkeys with green fluorescent protein (GFP)-tagged *P. cynomolgi* and fed mosquitoes with the blood obtained during peak parasitemia [68]. Sporozoites were harvested from the mosquitoes, and hepatocytes from macaque monkeys were infected using an in vitro system. These cells were sorted on the basis of GFP signal into hypnozoites (GFP_low_ signal) and schizonts (GFP_high_ signal), before RNA-seq was performed, and data from each group were compared

The study [66], although comprehensive, lacked data from one of the most interesting stages, the hypnozoite. Recently, several RNA-seq studies of hypnozoite-stage parasites have been performed, using either *P. vivax* [67] or a related simian parasite, *P. cynomolgi* [68] (Fig. 3). These dormant, liver-stage parasites are thought to be an adaptation to climates in which mosquitoes may not be present all year long, allowing the infection to persist for months or even years [69]. In humans, hypnozoites develop after infection with *P. vivax* and *P. ovale* parasites and can cause relapsing malaria. This stage of the parasite life cycle is challenging to study in humans because it is asymptomatic and is not detectable by blood tests or imaging studies. Thus, not much is known about the biology and pathophysiology of this stage. In addition, the only drugs that eliminate hypnozoites are the 8-amino quinolines primaquine and tafenoquine, both of which require the enzyme glucose-6-dehydrogenase (G6PD) for metabolism. Their mechanism of action remains unknown, and thus, there are no known targets for anti-relapse drugs and there is minimal understanding of the resistance to these therapies. In such situations, proteomics or transcriptional profiling might be used to find potential targets.

The Bhatia group used a previously developed, micropatterned primary human hepatocyte co-culture (MPCC) system to support the growth of *P. vivax* hypnozoites [70]. To complete the transcriptional profiling of *P. vivax* hypnozoites [67] (Fig. 3a), the authors extracted total RNA from *P. vivax*-infected MPCC cells and then enriched this RNA sample for *P. vivax* RNA using custom-made baits that tiled the recently assembled *P. vivax* P01 genome [71]. Cultures were enriched for hypnozoites by treating with a PI4K inhibitor, and the RNA-seq profile was compared to that of parasites from cultures that did not undergo PI4K treatment. Among the genes that were found to be transcribed in *P. vivax* hypnozoites, 40% encoded proteins of unknown function, whereas several genes found were important in metabolic and catalytic activity. The comparison revealed a decrease in the transcription of genes involved in processes such as maturation and merozoite invasion and egress in the hypnozoite-enriched samples. Particular members of the apicomplexan Apetala2 (ApiAP2) transcription family, which regulate parasite development [72], were upregulated in the hypnozoite-enriched samples. Furthermore, two known antimalarial drug targets, PI4K and eEF2, demonstrated decreased relative expression in the hypnozoite-enriched samples. The authors were also able to configure the MPCC system into a 384-well format to enable future high-throughput screening.

Voorberg-van der Wel and colleagues [68] infected rhesus monkeys with a green fluorescent protein (GFP)-tagged *P. cynomolgi* strain, fed mosquitoes with the infected blood, and then harvested the sporozoites from the infected mosquitoes and used these sporozoites to infect hepatocytes from macaque monkeys (Fig. 3b). These hepatocytes were then sorted on the basis of high versus low GFP signal, with low GFP signal representing hypnozoites, which enabled the transcriptional profiling of *P. cynomolgi* hypnozoites [68]. Although transcripts of several known drug targets were detected, their expression did not correlate with the activity of these drugs against liver-stage hypnozoite parasites. PI4K transcripts were expressed in the schizonts but not in the hypnozoites. Both studies [67, 68] reported a low number of detectable transcripts in the hypnozoite stage, with the most abundant transcripts mapping to genes that also had abundant transcripts in other life cycle stages (for example, histone-encoding transcripts). If hypnozoite-specific transcripts are to be identified more precisely, it is likely that much higher depth sequencing coverage will be needed. Nevertheless, with improvements in high-throughput phenotypic screening methods [67, 73], it should be possible to begin to identify compounds that target the hypnozoite stage and to then start working backwards from these.

Although less related to drug discovery, single-cell sequencing approaches could theoretically be used to understand compound mechanisms of action. One challenge with using studies of transcription to understand a drug’s mechanism of action is that it can be difficult to decide which parasite stage to examine. Single-cell sequencing should overcome this issue. Studies of the cultured asexual blood stage of *P. falciparum* [74] and of patient samples [75] have demonstrated the feasibility of this approach, although sequencing coverage remains lower than ideal.

## Parasite epigenomic landscape

It is notable that IVIEWGA approaches have yet to identify mutations that clearly confer drug resistance by increasing or decreasing target transcript levels, even though hundreds of intergenic mutations have been identified in various published genome scans of isogenic drug-resistant lines [32]. Until recently, a challenge in assessing the importance of intergenic mutations was that no data were available to indicate whether a mutation was in a possible regulatory region. Recently, however, a study using the assay for transposase-accessible chromatin using sequencing (ATAC-seq) approach was performed on *P. falciparum* intraerythrocytic stages, identifying 4000 regulatory regions [76]. Toenhake et al. [76] were able to show that these accessible regions encode regulatory regions by determining that these regions are enriched for sequence motifs that are known to control transcription. The authors were also able to rediscover motifs originally discovered by gene expression analysis [77], several of which (for example, PfM18.1 and PfM24.1) have been matched to transcription factors (AP-I [78] and AP-SP [79], respectively). In addition, the PfM18.1(GTGCA) motif—which had been linked to the expression of *P. falciparum* red cell invasion genes through de novo searching of transcriptional data [77] (with a log_10_ probability value of − 11.88)—was rediscovered in the ATAC-seq data (PF3D7_1007700_D3, with a log_10_ *p* value of − 5.94). Chromatin immunoprecipitation sequencing (ChIP-seq) studies have shown that this motif is the binding site for the AP-I transcription factor [78]. The smaller probability of enrichment by chance from the gene expression data alone is probably due to the fact that the gene expression data that were originally used covered the entire *P. falciparum* life cycle, including gametocytes and sporozoites, rather than just the asexual blood stage. This highlights how important it is to collect and include data from throughout the parasite’s life cycle, as with the *P. vivax* hypnozoite studies [67]. It is just as important to know when a gene is not expressed as when it is expressed.

Another interesting dataset that will help with the interpretation of whole-genome sequence data was that provided by Fraschka et al. [80]. To map out genome regions that are transcriptionally silent, this group profiled genome-wide heterochromatin protein 1 (HP1) occupancy in multiple *Plasmodium* species using ChIP-seq. Heterochromatin is marked by trimethylation of lysine 9 on histone H3 (H3K9me3) and binds to HP1, a regulator of heterochromatin formation and gene silencing [81]. Fraschka et al. [80] showed that, although the *Plasmodium* heterochromatin landscape is reproducible and primarily limited to the subtelomeric regions that are home to multigene families involved primarily in immune evasion, this landscape changes across parasite lines and species as well as during development. For example, significant changes were seen in heterochromatin binding between the asexual blood stage and the sexual gametocyte stage of *P. falciparum*. Silencing for certain gametocyte-specific transcripts was lost as the parasites matured into gametocytes. Although genes that are located in heterochromatin are unlikely to be drug targets (for example, they may not be expressed and therefore may not be critically essential), the map provided by this work will be useful in the search for new ways to limit parasite growth.

## Host transcription

If a compound with antimalarial activity acts against a human target, knowing which host genes are transcribed during infection may also provide hints about the compound’s possible target. There have been recent advances in understanding the human transcriptional response to parasite infection in liver stages [82, 83]. When parasites invade a human liver cell, a parasitophorous vacuole is formed. The parasites undergo many rounds of DNA replication during which host cell division is halted. It is likely that the host transcriptome is altered to feed the developing parasite and to avoid recognition by the immune system. Each of the upregulated host genes, if essential for parasite development, could be a possible target for drugs that prevent the parasite from developing further. Several previous studies sought to identify parasite genes that are turned off or on during hepatic-stage development [84]. In addition, a few studies examined the host response using microarrays, although the reported changes were modest [85]. Recent studies have examined the host transcriptome with RNA-seq [82, 83]. In both of these RNA-seq studies, the authors flow-sorted a variety of hepatoma lines that had been infected with GFP-labeled *P. berghei* and compared the host cell response to that in sorted, uninfected sister cells by performing extensive RNA-seq analysis. A major difference in the results was that one study identified thousands of differentially expressed human genes over the developmental time course [83] whereas the second study revealed relatively few statistically significant changes [82]. Nevertheless, the changes that were observed in the second study were validated and extensively characterized, and the authors convincingly showed that human aquaporin 3 was upregulated in response to parasite infection [82].

Dual RNA-seq studies (on both the host and the parasite) have also been performed on blood samples from symptomatic malaria patients. Lee et al. [86] performed dual RNA-seq transcriptome analyses on 33 samples from 46 *P. falciparum*-infected Gambian children. These authors performed whole-blood dual RNA-seq and identified a set of both human and parasite genes that demonstrated significant differential expression between subjects with uncomplicated or severe malaria. They found that the human genes encoding neutrophil granule proteins had the most differential expression, with high expression consistently associated with severe malaria. A general concern with performing transcriptional analysis on a limited number of patients is that the sample sizes may be too small to demonstrate significant findings considering the potential underlying genomic differences in the parasites. In this case, the differences in parasite gene expression between the severe group and the uncomplicated group were mild at best. On the other hand, the one parasite gene that consistently showed the strongest difference in gene expression between parasites in severe and uncomplicated malaria was *GBP130* (PF3D7_1016300), which encodes a possible glycophorin binding protein [87]. In *P. falciparum*, this gene is highly polymorphic with multiple repeats of the Pfam glycophorin-binding domain but is nonessential [88]. This parasite protein is predicted to bind to a member of the glycophorin family, the human-encoded family of invasion receptors for *P. falciparum* [89]. Structural variants in the human genome surround glycophorin-encoding genes [90] (as noted below), and some of these structural variants are associated with protection from severe malaria.

## Host genetics

Molecules that disrupt the growth of parasites within human cells (either red blood cells or infected hepatocytes) could theoretically target human cells. Thus, knowledge of which human gene products are needed by the parasite in order to gain access and grow could inform target deconvolution. Given the high mortality rate from untreated severe *P. falciparum* infections [91], malaria would be expected to have a strong effect on the human genome, with selective sweeps or linkage disequilibrium evident in the genomes of humans living in malaria-endemic regions. Most previous discoveries have focused on single-nucleotide variants, which are easier to detect, but advances in the detection of structural variants in the human genome have contributed to the discovery of novel malaria resistance alleles. Genome-wide association studies had previously discovered a region on chromosome 4 that was associated with resistance to severe *P. falciparum* malaria, although no causative alleles were identified [92]. The Malaria Genomic Epidemiology Network carefully sequenced this region and discovered a series of structural variants affecting the parasite invasion receptor genes *GYPA* and *GYPB* (encoding glycophorin proteins), both located near the region associated with human resistance to severe malaria mortality [90]. One complex variant, the Dantu blood group variant, reduces the risk of severe malaria by 40%, and the frequency of this variant in the population has recently increased in parts of Kenya. It would be interesting to determine whether there is an association between disease severity, human Dantu blood group phenotype, and parasite GBP130 expression or genotype. It is likely that future studies to test such an association will need to be performed with parasite lines taken recently from the field, as well as with human donors with different red blood cell groups. These data also highlight how parasite evolution and human evolution may be occurring concurrently.

Previously, a candidate gene approach was used to show that alleles of a gene that is involved in sensing motion may contribute to malaria susceptibility in human populations [93]. Family mapping studies aiming to identify the genetic basis of hereditary xerocytosis, a red blood cell disorder, identified a candidate region on chromosome 16 encompassing the gene encoding the mechanotransduction protein PIEZO1 [93]. As many red cell disorders (for example, sickle cell disease) confer resistance to malaria, Ma et al. [94] introduced the PIEZO1 allele (R2482H) associated with human xerocytosis into mice. The gene-edited mice were less susceptible to severe malaria when infected with the rodent parasite *P. berghei* and survived longer. Ma et al. [94] next searched human populations for additional mutations in PIEZO1 and discovered an E756del PIEZO1 allele in African populations (present in 18% of individuals of African descent). Functional studies showed that this allele caused statistically significant changes in signal transduction, and *P. falciparum* growth assays performed with donated human red cells harboring the E756del allele revealed that these cells supported less *P. falciparum* growth [94], suggesting that the allele may protect against *P. falciparum* malaria in human populations. Further work will be needed to test for an association with protection from severe malaria in endemic regions.

## Advances in target identification in other protozoan pathogens

Target validation approaches are well established for malaria parasites, and these approaches have also been extended to other protozoan pathogens, including *Trypanosoma*, *Leishmania*, and *Toxoplasma*. *Trypanosoma* parasites are kinetoplastids that cause Chagas disease (*T. cruzi*) [95] and sleeping sickness (*T. brucei*) [96]. Chagas disease is treated with the nitroheterocyclic drugs nifurtimox and benznidazole, whose mechanism of action is not well understood but is thought to involve oxidative stress [4] (Table 1). These medications do not have activity against the chronic stages of infection, require a prolonged course of treatment, and have several adverse effects. Treatments available for sleeping sickness include suramin, melarsoprol, pentamidine, and eflornithine [5]. These medications must be given intravenously or intramuscularly, and have many toxic side effects. In addition, there is clinical evidence of resistance to melarsoprol [5]. *Leishmania* parasites can cause cutaneous disease with severe soft tissue infections, visceral disease with systemic illness with organ involvement, or mucosal disease with primarily mucous membrane involvement. Leishmaniasis is primarily treated with pentavalent antimonial compounds, liposomal amphotericin B, paromomycin, and miltefosine. These medications have a high cost, limited efficacy, and toxic side effects. In addition, *Leishmania* strains have demonstrated antimonial resistance and species-dependent variations in drug susceptibility [8]. *Toxoplasma* parasites can cause a flu-like illness in immunocompetent hosts, a disseminated infection with ocular and central nervous system involvement in immunocompromised hosts, and congenital infection with severe manifestations. Similar to *Plasmodium*, *Toxoplasma* spp. are apicomplexan parasites. Treatment is with pyrimethamine and sulfadiazine. These medications require a prolonged course of therapy and can have severe adverse effects, and there are reports of treatment failures possibly resulting from drug resistance [12, 13]. Progress and challenges in drug discovery and development for *Trypanosoma*, *Leishmania*, and *Toxoplasma* parasites have been reviewed elsewhere [6, 9, 13], but a few recent advances involving omics-based methods are highlighted here.

As in studies of malaria, an established way to identify chemically validated targets in other protozoan pathogens is to start with a compound that has attractive cell-killing properties and to work backwards. For *Trypanosoma* and *Leishmania*, phenotypic screens have led to the identification of the most promising drug targets, whereas target-based approaches have been less successful overall, with few strong drug targets identified [6]. Wyllie et al. [43] first identified and partially optimized a pyrazolopyrimidine compound that has both cellular and organismal activity against *Leishmania donovani*, the causative organism in visceral leishmaniasis. They then used a combination of chemical proteomics and IVIEWGA to identify cyclin-dependent kinase 12 (CDK12) as the target. The work was remarkable in that genome analysis in *Leishmania* is more complex than that in malaria parasites owing to the larger and diploid genome. Indeed, the analysis of the sequenced clones showed more mutations than ideal, but because some of the top hits were also found in proteomic analyses, it was straightforward to select likely candidates [43]. CDK12 will now become an attractive target for structure-guided drug discovery.

Khare et al. [41] also used whole-genome analysis to assess whether the proteasome was the target of GNF3943, a predicted proteasome inhibitor. The lead compound was identified using a phenotypic screen for compounds that are broadly active against *Leishmania donovani*, *Trypanosoma cruzi*, and *Trypanosoma brucei*. The authors then synthesized around 3000 compounds with the goal of improving both bioavailability (using a mouse model) and inhibition of *L. donovani* growth within mouse macrophages and selected two for IVIEWGA experiments. Whole-genome sequencing of a GNF3943-resistant line showed that this line bears a homozygous mutation that results in an isoleucine-for-methionine substitution at amino acid 29 in the proteasome β4 subunit (PSMB4; I29M). Sequencing of a resistant line from a closely related compound, GNF8000, identified another mutation (F24 L) in PSMB4. The identification of these two independent mutations suggested that the proteasome was the probable target of the compound series because the proteasome is essential in eukaryotic cells. Of note, the proteasome has also been detected as a promising target in *P. falciparum* [30], and IVIEWGA has been used recently to confirm on-target activity for derivatives of bortezomib, a proteasome inhibitor [25], and for TCMDC-135051, an inhibitor of *P. falciparum* cyclin-dependent-like kinase 3 (CLK3) [40].

Leads for drug discovery in *Toxoplasma* have also been identified primarily through the phenotypic screening of compounds using well-established in vitro or in vivo systems [13]. One particular challenge with *Toxoplasma* is the lack of an in vitro system for high-throughput screening of compounds against the cyst-forming bradyzoite phase, which must be studied in vivo. Methods for determining the mechanism of action of the compounds that are identified from high-throughput screens are not as well developed in *T. gondii* compared to other protozoan pathogens, but Amberg-Johnson and colleagues [97] recently used IVIEWGA in *T. gondii* to discover that the membrane metalloprotease FtsH1 is the target of an antimalarial compound.

Genome-wide essentiality screens have also identified potentially druggable pathways. Sidik et al. [45] used a CRISPR-Cas9 system to identify *T. gondii* genes that are required during the infection of fibroblasts. They found 17 indispensable conserved apicomplexan proteins (ICAPs), 8 of which were localized to the mitochondria. One of the proteins, ICAP12, which was structurally similar to mammalian tight junction claudin proteins and was named claudin-like apicomplexan microneme protein (CLAMP), was found to be essential for the invasion of host cells. The knockdown of its ortholog in *P. falciparum* completely inhibited the asexual blood stage. This study demonstrated the utility of the CRISPR-Cas9 system in developing a baseline understanding of gene essentiality in apicomplexans, but it did not take into account the changes resulting from additional pressures such as the immune response or the life cycle stage transitions that occur in actual infections.

## Structure-guided drug discovery and exceptions

Although targets that are discovered using IVIEWGA often have clearly identifiable binding sites for small molecules (for example, they may have an ATP-binding motif), with enough knowledge and perseverance, it may be possible to inhibit other essential proteins. Structure-guided drug discovery is a type of target-based approach that is used for hit-to-lead optimization for the identification of potential small molecule binding sites, where small molecules are screened against a purified target protein, such as an essential enzyme. In *Trypanosoma* spp., glycosomes are essential organelles that are required for glucose metabolism and whose biogenesis is dependent on peroxins (PEX) [98]. Dawidowski et al. [42] used an elegant nuclear magnetic resonance (NMR)-based structure-guided drug discovery approach to identify small molecules that disrupt a key PEX14-PEX5 protein-protein interaction in *T. cruzi*. This interaction is essential for glycosomal biogenesis and import [99]. The study [42] was a remarkable achievement as there is a virtually unlimited number of protein-protein interactions in the cell, and targeting interactions greatly opens up the number of potential inhibitor-binding locations. However, despite moderate in vitro activity, the authors were not able to achieve a significant reduction in parasitemia. The rational challenge with structure-guided approaches is that success ultimately depends on whether the target is a good one and whether inhibition will ultimately lead to a cure in an animal model of disease. Although inhibitors may be designed that are on-target and that have a potent inhibitory effect in vitro, it may be impossible to achieve a cure in an animal model of disease if the activity is not potent enough in vivo. The lack of cure could be the result of pharmacokinetic issues and problems with delivery, but the possibility remains that the target may simply not be as critical in vivo as was observed during in vitro studies. In this case, no amount of optimization would yield a molecule with curative potential. However, if a good target is identified and used for structure-guided drug design, the probability of achieving a cure in an animal model is improved, as shown by recent efforts against *P. falciparum* lysyl tRNA synthetase [100], a target discovered using IVIEWGA 2 [101].

## Conclusions and future directions

The application of genomics and omics-based methods has enabled notable advances in the identification of novel targets in protozoan pathogens, and we have highlighted some of the advantages and disadvantages of these technologies (Table 2). In particular, cell-based phenotypic compound screening has facilitated the discovery of antimalarial drug targets for different parasite stages. The forward genetics IVIEWGA method has been one of the most successful omics-based methods used for discovering or rediscovering many specific novel targets of promising small molecules. Some of the promising novel antimalarial targets identified include proteasome subunits, eEF2, PheRS, cytoplasmic IRS, lysyl tRNA synthase, PfATP4, dihydroorotate dehydrogenase, and cytochrome bc1 (see [17] for a review). Many of the compounds that inhibit these targets demonstrate potent activity during multiple life cycle stages.

For the most part, the targets that have been discovered using forward genetics approaches fall into protein classes that were known to be druggable in other species. Many have ATP-binding sites or pockets that accommodate small molecules. Nevertheless, just because a protein is found to be essential for growth in an omics-based assay, this does not mean that it may be druggable—for example, it may not have binding sites for a small molecule, it may not be critically essential, or its cellular levels may be so high that its function cannot be disrupted at pharmacologically relevant inhibitor concentrations. As the in vitro evolution approach begins with exposure to small molecule compounds that are drug-like, it is more likely that this approach will identify druggable targets. On the other hand, some researchers are now considering the possibility of using therapeutic monoclonal antibodies for long-term control or prevention of parasitic infections [102]. If this method gains traction, other targets discovered using omics-based approaches might become more interesting—for example, a therapeutic antibody might be developed that inhibits glycophorin binding.

Interestingly, conservation across pathogens also seems to be observed. The proteasome represents a promising drug target for malaria parasites, as well as for *Trypanosoma* and *Leishmania* species [17, 41], as does cytochrome bc1 [17, 103]. In addition, tRNA synthetases are good targets in multiple species, as tRNA synthetase-targeting compounds that are active in malaria are also active in cryptosporidiosis [100]. Other promising targets include CDK12 for *Leishmania* [43]. Many of the most promising of these targets also have human analogs, indicating that the design of selectivity for the parasite targets will be a very important step in the long process of drug development. Nevertheless, optimization of the compounds to minimize host toxicity and to ensure robust in vivo activity is clearly an achievable goal. For example, tavaborole (AN2690) is a new FDA-approved treatment for fungal infections which targets leucyl-tRNA synthetase, a target that was discovered using IVIEWGA [104].

Although IVIEWGA has led to a number of successes, it is not without limitations. Problems include distinguishing between resistance genes and targets, an inability to generate resistance to some compounds, the reappearance of resistance genes, and the lack of methods that enable target discovery for compounds that are not active in malaria parasite blood stages. In these cases, proteomic methods may be more important. Genome-wide over- and underexpression libraries, which have been used for target deconvolution in other pathogens such as *Mycobacteria tuberculosis* [105], could also prove useful. Arrayed CRISPR-Cas9 disruption libraries could theoretically help to identify human targets that are essential for parasite growth and development. In addition, a recent RNAi knockdown screen of the human druggable genome identified secretion factors as critical for parasite development in human liver cells [106].

Once important genes are discovered using forward genetics methods, additional biological work is needed to determine how alleles confer resistance, which is where functional genomic methods can play an important role. Databases such as PlasmoDB [88], which display phenotypes in addition to gene- and protein-level data, are invaluable when making a decision about which genes deserve follow-up. Databases are also very useful when interpreting mass spectrometry data.

A common question is whether or not issues of drug resistance will remain problematic for targets discovered using IVIEWGA. In theory, it might be possible to design inhibitors that a pathogen is less able to acquire resistance against, but this could be chemically challenging. For now, compounds that come from this strategic approach will most probably need to be deployed as a component of combination therapies in order to avoid the development of drug resistance.

The rapid growth and falling costs of omics-based technologies have led to their applications in studies of protozoan pathogens that have revealed promising new drug targets in addition to new insights about parasite biology. The development of new drugs for these important pathogens is of increasing importance as the threat of drug resistance continues to grow. However, as highlighted above, the findings of these recent studies require further follow-up exploration and testing to elucidate or confirm potential drug targets.

## Abbreviations

ATAC-seq: Transposase-accessible chromatin using sequencing
ChIP-seq: Chromatin immunoprecipitation sequencing
eEF2: Elongation factor 2
FDA: Food and Drug Administration
GFP: Green fluorescent protein
HP1: Heterochromatin protein 1
IRS: Isoleucine tRNA synthase
IVIEWGA: In vitro evolution and whole-genome analysis
MFS: Mutant fitness score
MPCC: Micropatterned primary human hepatocyte co-culture
PfATP4: P-type cation-ATPase 4
PfEMP1: *P. falciparum* erythrocyte membrane protein 1
PheRS: Phenylalanine tRNA synthetase
PI3P: Phosphatidylinositol-3-phosphate
PI4K: Phosphatidylinositol-4-kinase
